# Genetic and Phenotypic Characterization of Domestic Geese (*Anser anser*) in Egypt

**DOI:** 10.3390/ani11113106

**Published:** 2021-10-30

**Authors:** El-Sayed M. Abdel-Kafy, Sherif I. Ramadan, Weal H. Ali, Sabbah F. Youssef, Hoda A. Shabaan, Amira El-Deighadi, Miho Inoue-Murayama

**Affiliations:** 1 Agricultural Research Center (ARC), Animal Production Research Institute (APRI), Dokki, Giza 12651, Egypt; sayedabdkaffy@yahoo.com (E.-S.M.A.-K.); waha200@yahoo.com (W.H.A.); Sabbah.farouk@yahoo.com (S.F.Y.); hodamshabaan@yahoo.com (H.A.S.); aboelfadleziadmagdy@gmail.com (A.E.-D.); 2 Department of Animal Wealth Development, Faculty of Veterinary Medicine, Benha University, Toukh 13736, Egypt; 3 Wildlife Research Center, Kyoto University, Kyoto 606-8203, Japan; mmurayama@wrc.kyoto-u.ac.jp

**Keywords:** base population, body measurements, Egyptian geese, mtDNA, microsatellite

## Abstract

**Simple Summary:**

The production of domestic geese in Egypt depends mainly on small-sized flocks reared by smallholder farmers in villages, and until now, there have been no intensive or commercial goose farms in Egypt. The objective of this study was to characterize three domestic Egyptian goose populations (Kafr El-Sheikh, Fayoum and Luxor) phenotypically and genetically in order to identify the populations with the highest diversity to establish a large base population with a broad variation. Phenotypic characterization of 402 domestic mature geese included morphological measurements such as head length, culmen length, bill width, tarsus length, sternum length and chest circumference. Genetic characterization of 173 individuals was performed based on mutations in the mitochondrial D-loop region and the genotyping of 12 microsatellite markers. The results showed low population differentiation based on morphological measurements and low genetic differentiation based on the two used genetic markers. The low differentiation between the three investigated goose populations implies their suitability for aggregation and formation of a large founder population with high genetic variation. The information from this study could be useful for further investigation in order to develop a convenient conservation program for this important species.

**Abstract:**

The objectives of this study were to achieve phenotypic characterization of three domestic Egyptian goose populations collected from three different geographical zones (Kafr El-Sheikh, Fayoum and Luxor) and to perform genetic characterization of these three populations based on mtDNA D-loop and 12 microsatellite markers. The body measurements of 402 domestic mature geese belonging to these three governorates showed that the lengths of the head, culmen and tarsus and the live body weight varied significantly among the three studied Egyptian goose populations. After alignment of a 710-base-pair segment of the goose mtDNA control region, there was a single haplotype in the three Egyptian goose populations, indicating the same maternal origins. The genotyping of the 12 microsatellite markers showed low diversity indices, including average observed (*N_A_*) and effective (*N_E_*) number of alleles and observed (*H_O_*) and expected heterozygosity (*H_E_*) (3.333, 1.760, 0.277 and 0.352, respectively), and a high inbreeding coefficient (*F_IS_* = 0.203) across the three Egyptian goose populations. The high inbreeding and low genetic and morphological differentiation of Egyptian geese could be corrected by establishing a large base population through capturing small populations with the highest genetic variation. The findings of the current study can therefore serve as an initial guide to design further investigations for developing conservation programs of Egyptian geese genetic resources.

## 1. Introduction

Globally, domesticated geese were derived from two wild species: (1) the greylag goose (*Anser anser*), a progenitor of many domestic breeds, including the current native geese in Egypt; and (2) the swan goose (*Anser cygnoides*), an ancestor of the Chinese and African goose breeds [1,2]. Historically, the goose was one of the first birds domesticated in Egypt more than 3000 years ago [3,4,5,6]. It was used for meat, fat and down, as well as in the cultic sphere [6]. Ancient Egyptians invented a force-feeding technique for producing fatty liver (around 2686–2181 BC) and introduced the feather plucking technique of geese [7,8].

The drawings of geese on the walls of ancient Egyptian temples showed the two types of domesticated geese in Egypt [9]. The first one was the Egyptian goose (*Alopochen aegyptiaca*) [6,9,10], a wild species belonging to the subfamily Tadorninae and considered the only extant member of the *Alopochen* genus [9]. This species was domesticated in the Old Kingdom that ended around 2300 BC. Further breeding of this very common domesticated species, however, stopped after the Persian conquest of Egypt in 525–524 BC [2]. The second type of domesticated geese descended from the greylag goose (*Anser anser*) [6]. It is known that there was an extraordinarily large goose breed in ancient Egypt in ~600 BC to 200 AD [7,8,10,11].

Considering the established goose breeding and production practices in ancient Egypt, some authors [4,5] single out an Egyptian center of domestication, breed formation and dispersion of domestic geese, among six such centers in the world.

The domestic Egyptian geese are characterized by barred grey and white feathers, with the legs, feet and beaks ranging from orange to pink colors [12]. Geese in Egypt are presently reared for many purposes, such as a source of eggs, meat and as guarding, especially in Upper Egypt [13]. The production of domestic Egyptian geese depends mainly on small-sized flocks reared by smallholder farmers in the villages around the Nile valley that could be classified into three major regions, namely, the Upper, Middle and Delta Egypt regions, and there are currently no intensive or commercial geese farms in Egypt [9,14]. Thus, the establishment of a large base population with a broad genetic variation of domestic Egyptian geese is eagerly anticipated.

High genetic variation is a very important factor for establishing a successful base population as the response due to selection depends mainly on additive genetic variation. One method for maximizing the genetic variation in the base population is to aggregate and capture small-sized populations with the highest genetic diversity from different breeders into a large founder breeding population [15,16,17]. Phenotypic and genetic characterization of these small-sized populations will help in optimizing and capturing the populations with the highest genetic variation to be used while establishing the base population.

There are several methods used to characterize poultry diversity, ranging from assessment and linear measurement of morphological traits [18,19,20,21] to the use of molecular techniques [22]. Morphological measurements have been used to characterize and compare various geese species and populations [13,23,24,25], while microsatellites and mtDNA have been used for genetic characterization and evaluation of phylogenetic relationships among various geese populations [26,27,28]. Domestic Egyptian geese are still poorly characterized in terms of phenotypic and genetic aspects [12,22,28]. Further investigations are needed to shed light on the remarkable genetic potential of this important species [29], especially in countries of traditional goose keeping, such as Egypt [6], to improve their breeding on farms and in courtyards [29,30].

Therefore, the objectives of this study were to perform phenotypic characterization of three domestic Egyptian goose (*Anser anser*) populations collected from three different geographical zones and also to perform genetic characterization of these three populations based on the mtDNA D-loop and 12 microsatellite markers. This information will help in optimizing the contribution of each population while creating a large base population of Egyptian geese.

## 2. Materials and Methods

### 2.1. Statement of Animal Rights

The current study was conducted according to protocols that were approved by the Committee of Animal Care and Welfare, Animal Production Research Institute, Egypt, in June 2016 (ethical approval number: 020203429).

### 2.2. Sampling Sites

**Figure 1:**
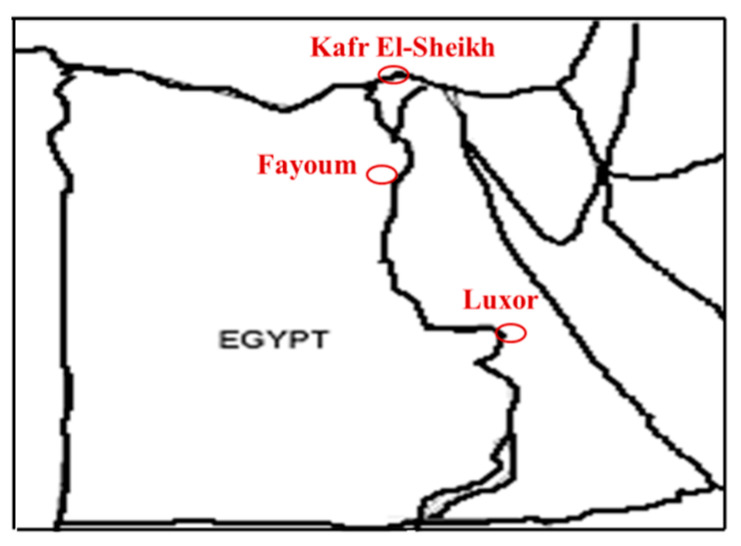
Location of the Kafr El-Sheikh, Fayoum and Luxor governorates in Egypt.

This study was carried out in three geographical zones: the Upper, Middle and Delta Egypt regions, as shown in Figure 1.

Upper Egypt was represented by 12 villages located in the Luxor governorate, Middle Egypt was represented by 13 villages located in the Fayoum governorate and Delta Egypt was represented by 13 villages located in the Kafr El-Sheikh governorate, as shown in Table 1. The survey was carried out by the Animal Production Research Institute (APRI) team by organizing visits to smallholders of domestic Egyptian geese within these three governorates.

### 2.3. Body Measurements

The body weight and body measurements of 402 domestic mature geese with an equal sex ratio were estimated according to the procedures of [31]. Body measurements included head length, culmen length, bill width, tarsus length, sternum length and chest circumference, as shown in Supplementary Figure S1. Body measurements were determined by using diagonal calipers and a metric ruler. In order to avoid inter-individual variation, all measurements were carried out by the same person.

### 2.4. Samples for DNA Extraction

**Figure 2:**
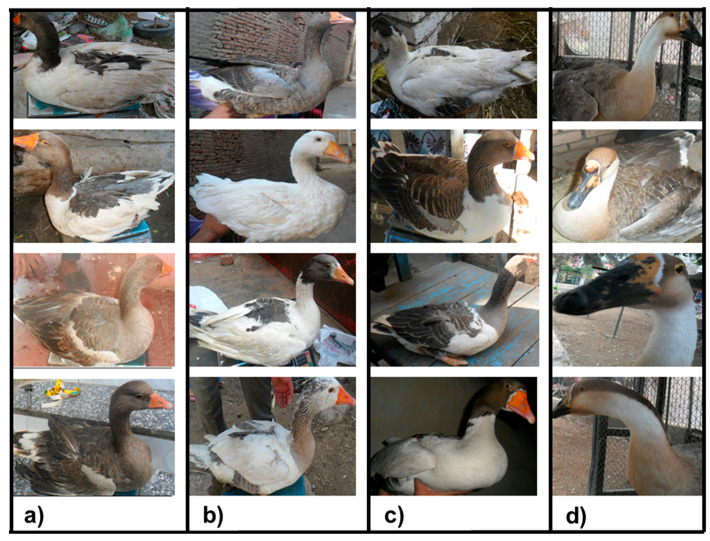
Collected photos of the native Egyptian—(**a**) Kafr El-Sheikh, (**b**) Fayoum and (**c**) Luxor—and (**d**) Chinese geese populations.

**Figure 3:**
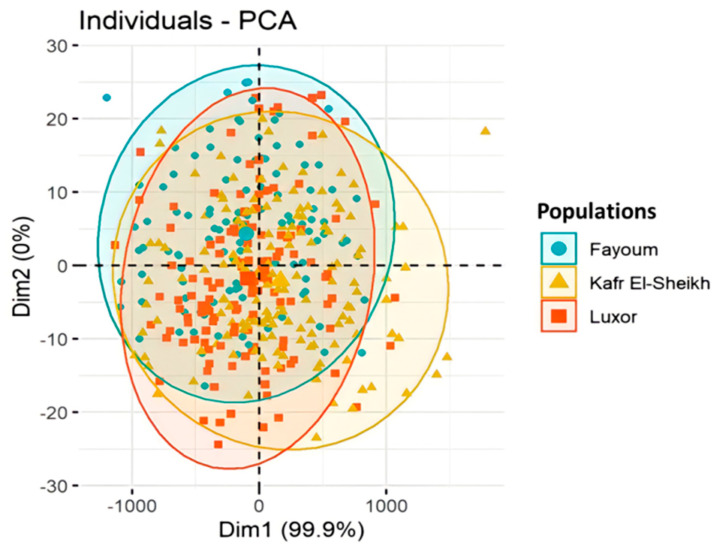
Plot of the principal components PC1 (Dim1) and PC2 (Dim2) and factor loadings from the principal component analysis (PCA) of body measurements in the three Egyptian goose populations.

Blood samples were taken from 173 individuals selected randomly out of the above 402 domestic geese belonging to the three governorates (Kafr El-Sheikh, Fayoum and Luxor). In addition, 31 samples from Chinese geese (*Anser cygnoides*) in the zoos located in the Kafr El-Sheikh and Giza governorates were collected. Collected photos for the three studied Egyptian and Chinese goose populations are shown in Figure 2 and Figure 3. DNA was extracted by using a GeneJET Genomic DNA Purification Kit following the manufacturer’s protocol (#K0721, Fermentas, Waltham, MA, USA). Blood samples were taken from the wing vein of the animals and stored at −20 °C.

### 2.5. Mitochondrial D-Loop Analysis

PCR was performed to amplify a 710-base-pair segment of the goose mitochondrial control region (D-loop) by using the primer pair F: 5′-CCTCTGGTTCCTCGGTCA-3′ and R: 5′-CAACTTCAGTGCCATGCTTT-3′ [32]. Two samples from Egyptian domestic geese from each governorate were amplified and sequenced. Moreover, one sample from a Chinese goose (*Anser cygnoides*) was sequenced for comparison. PCR was performed in a total volume of 15 µL, containing 20 ng of mtDNA, 2× PCR buffer, 400 μM of each dNTP, 0.3 μM of each primer and LA-Taq polymerase at 0.5 U (Takara, Shiga, Japan). PCR amplification was performed for 35 cycles after initial incubation at 95 °C for 5 min. Each PCR cycle consisted of 95 °C for 45 s, an annealing temperature of 56 °C for 45 s, 72 °C for 60 s, followed by a final extension of 72 °C for 10 min. A PCR purification kit (Roche, Mannheim, Germany) was used for purification of the amplified products; then, the purified products were sequenced using the same primers with the Big Dye Terminator ver. 3.1 Cycle sequencing kit (Applied Biosystems, Foster City, CA, USA) and electrophoresed on an ABI PRISM 3130xl DNA sequencer (Applied Biosystems, Foster City, CA, USA). BLAST software [33] was used for sequence identification and confirmation. MEGA 7 [34] was used for sequences alignment and to infer the phylogenetic relationships based on neighbor-joining methods [35].

### 2.6. Microsatellite Genotyping

Twelve microsatellite markers, namely, *ZAAS006*, *ZAAS013*, *ZAAS018*, *ZAAS035*, *ZAAS038*, *ZAAS015*, *ZAAS060*, *ZAAS064*, *ZAAS152*, *ZAAS041*, *ZAAS175* [36] and *ANS025* [37], with minimum fragment overlapping, were selected and amplified in three PCR multiplex reactions using the QIAGEN Multiplex PCR Kit (QIAGEN, Valencia, CA, USA). PCR was performed in a total volume of 10 µL, containing 20 ng of DNA template; 0.2 µM of each primer, of which the forward ones were fluorescently labeled (FAM and NED); and 2× QIAGEN Multiplex PCR Master Mix. PCR amplification was conducted for 35 cycles after initial incubation at 95 °C for 15 min. Each PCR cycle consisted of 95 °C for 30 s, an annealing temperature of 51–59 °C for 90 s, 72 °C for 60 s, followed by a final extension of 60 °C for 30 min. Then, the PCR products were electrophoresed on an ABI 3130xl DNA Sequencer (Applied Biosystems, Foster City, CA, USA), and based on the 400 HD ROX size marker, the sizes of the fragments were estimated using the GENEMAPPER software (Applied Biosystems, Foster City, CA, USA).

### 2.7. Statistical Analysis

The statistical difference between the three studied goose populations was assessed using a one-way analysis of variance (ANOVA), with the population of geese included in the model as the source of variation using the PROC GLM procedure in the SAS v9.1.3 statistical package (SAS Institute Inc., Cary, NC, USA). We conducted a principal component analysis (PCA) using R software version 3.6 (R Foundation for statistical computing, Vienna, Austria). A stepwise discriminant analysis was conducted using PROC STEPDISC to investigate which morphological traits have more discriminant power than others. The level of significance (*p <* 0.05) and partial R^2^ values of ≥0.01 were used to evaluate the relative importance of the morphometric variables in discriminating the three populations of geese.

Genetic diversity parameters were evaluated by calculating the observed (*N_A_*) and effective (*N_E_*) number of alleles, the observed (*H_O_*) and expected (*H_E_*) heterozygosity and the Hardy–Weinberg equilibrium using GENALEX version 6.0 [38]. Polymorphic information content (*PIC*) was estimated using CERVUS version 3 [39]. F-statistics (fixation coefficient of a subpopulation within the total population (*F_ST_*), fixation coefficient of an individual within a subpopulation (*F_IS_*) and fixation coefficient of an individual within the total population (*F_IT_*)) per locus and deviation from the Hardy–Weinberg equilibrium (*HWE*) were estimated by the GENEPOP version 3.4 program [40]. The POPULATIONS version 1.2.30 software (http://bioinformatics-org/~tryphon/populations/ /accessed on 26 June 2021) was used to construct the microsatellite phylogenetic tree of the three studied populations based on the Reynolds and Nei genetic distance (*D_A_*) by using the neighbor-joining (NJ) method [35].

The clustering pattern and the genetic structure of the studied populations were investigated using STRUCTURE software [41]. For each value of *K*, we conducted 50 runs with 50,000 iterations following a burn-in period of 20,000. CLUMPP software [42] was used for pairwise comparison of the 50 solutions of each *K* and outputted a mean of the permuted matrices across replicates after aligning the cluster membership coefficients of these replicates. Finally, the clustering pattern with the best ∆*K* value was graphically displayed for the selected *K* value using DISTRUCT software [43].

## 3. Results

### 3.1. Body Measurements

The mean values of live body weight and other body measurements in the three evaluated Egyptian goose populations are presented in Table 2. The head, culmen and tarsus lengths and the live body weight varied significantly among the three studied goose populations, while bill width, chest circumference and sternum length did not show significant differences. The result of the stepwise discriminant analysis is presented in Table 3. The six measured variables were found to be significant (*p* < 0.05 to *p* < 0.001). However, chest circumference chronologically followed by body weight, sternum length and head length had more discriminant power than the others, as revealed by their higher R^2^ and *F*-values.

**Figure 4:**
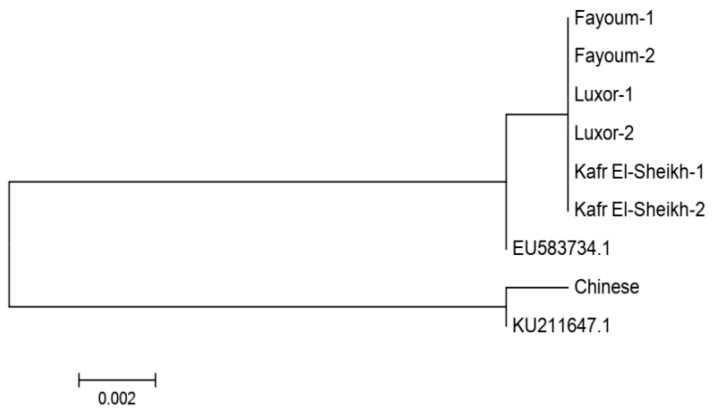
Neighbor-joining phylogenetic tree based on the mitochondrial D-loop sequence for three Egyptian goose populations. Accession numbers EU583734.1 and KU211647.1 were retrieved from GenBank for *Anser anser* and *Anser cygnoides*, respectively. The Chinese goose population was used as an outgroup.

The principal component analysis (PCA) of the body measurements showed that the first principal component (Dim1) accounts for 99.9% of the variation, while the second one (Dim2) accounts for 0.0% of the variation, as shown in Figure 4. Moreover, the collected photos of the three studied Egyptian goose populations showed unclear differences in the body measurements and feather colors, as shown in Figure 2a,b,c. In contrast, the collected photos of the Chinese geese (outgroup) showed great differences between the native and Chinese geese, as shown in Figure 2d.

### 3.2. Mitochondrial D-Loop Analysis

We obtained a sequence of 710 bp for two samples of each of the three Egyptian populations in addition to one sample of Chinese geese acting as an outgroup population. After alignment, there were no substitution sites among the three Egyptian goose populations, indicating a single haplotype and the same maternal origins. In the NJ phylogenetic tree, these three populations clustered into the same clade with the *Anser anser* sequence retrieved from GenBank (accession number EU583734.1), and the Chinese sample was clustered with the *Anser cygnoides* sequence retrieved from GenBank (accession number KU211647.1), as shown in Figure 4.

### 3.3. Microsatellite Marker Polymorphisms and Population Diversity

The genetic diversity of the 11 microsatellite loci across the three Egyptian goose populations is shown in Table 4. The number of observed alleles (*N_A_*) ranged between 2 (*ZAAS064*, *ZAAS152* and *ZAAS175*) and 7 (*ZAAS006* and *ZAAS018*), with an average of 3.333 alleles. The locus *ZAAS035* showed a monomorphic pattern across the three Egyptian and Chinese goose populations and was excluded. The effective number of alleles (*N_E_*) ranged from 1.024 (*ZAAS175*) to 2.748 (*ZAAS038*), with an average of 1.760 alleles.

The averages of the observed and expected heterozygosity (*H_O_*, *H_E_*) were 0.277 and 0.352, respectively. The *ZAAS152, ZAAS041* and *ZAAS175* loci showed very low observed (0.021, 0.041 and 0.024, respectively) and expected (0.034, 0.081 and 0.023, respectively) heterozygosity (Table 4). The mean fixation coefficient of an individual within a subpopulation (*F_IS_*) and the mean fixation coefficient of a subpopulation within the total population (*F_ST_*) were 0.203 and 0.045, respectively, across the eleven studied loci.

Although the *ZAAS152, ZAAS041* and *ZAAS175* loci showed very low *PIC* values (0.032, 0.077 and 0.23, respectively), the mean *PIC* value was 0.307. Significant deviations from the Hardy–Weinberg equilibrium (*HWE*) in allele frequencies were recorded for all markers except the *ZAAS015*, *ZAAS060* and *ANS025* loci (Table 4). Across the 11 loci, the average numbers of alleles and the expected and observed heterozygosity in addition to the *F_IS_* for each population are shown in Table 5.

The lowest value of expected heterozygosity (0.308) was obtained for the Kafr El-Sheikh population, and the highest value (0.343) was recorded for the Fayoum population. The overall expected heterozygosity of the three Egyptian populations was 0.325. The *F_IS_* value was calculated and found to range from 0.114 (Luxor) to 0.277 (Fayoum), with a mean of 0.213.

### 3.4. Genetic Relationship and Population Structure

**Figure 5:**
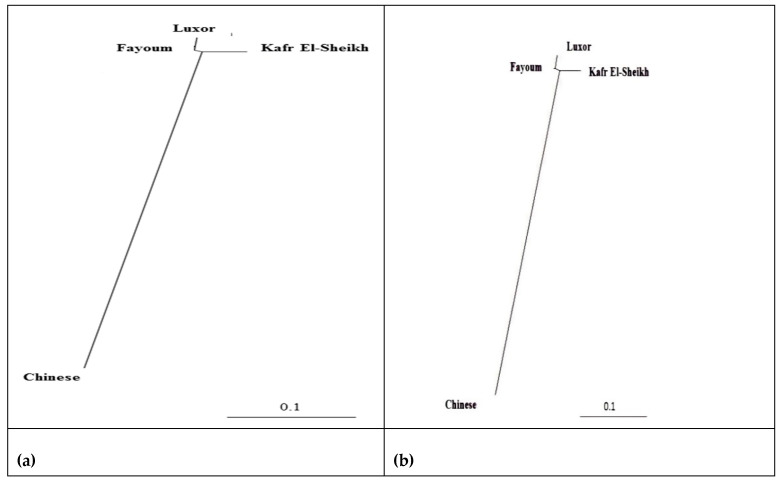
Neighbor-joining phylogenetic tree of the three Egyptian and Chinese goose populations by 11 microsatellite markers based on (**a**) Reynolds’ and (**b**) Nei’s genetic distances.

**Figure 6:**
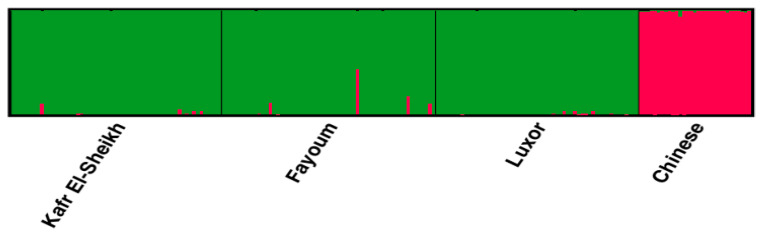
Structure clustering of the three Egyptian and Chinese populations obtained for *K* = 2.

The lowest pairwise Reynolds and Nei genetic distance (*D_A_*) value between the four studied goose populations was recorded between Fayoum and Luxor (0.023) and the highest value between the Kafr El-Sheikh and Luxor populations (0.071). Similarly, the genetic differentiation indicated by the pairwise *F_ST_* values was the lowest between Fayoum and Luxor (0.022) and the highest between Kafr El-Sheikh and Luxor (0.044), as shown in Table 6. These results are supported by the clustering pattern either from the neighbor-joining phylogenetic tree (Figure 5) or by the Bayesian clustering of STRUCTURE with the admixture method (Figure 6). The tree topology showed a close relationship between the Fayoum and Luxor populations. At *K* = 2, where the four studied goose populations showed the most probable structure clustering, the Fayoum, Kafr El-Sheikh and Luxor populations were clustered together, while the Chinese population was assigned independently into its respective cluster (Figure 6).

## 4. Discussion

### 4.1. Body Measurements

Characterization of the differences among family groups and closely related species of Egyptian geese requires the evaluation of body size and morphological measurements. The morphological measurements among the studied Egyptian geese showed values comparable to those described by Łukaszewicz et al. [44,45] in Canadian geese, who recorded ranges of 119 to 124, 92 to 99.4 and 52.3 to 56.3 mm for head, tarsus and culmen length, respectively, and by Łukaszewicz et al. and Nowicka and Przybylski [44,46], who reported values of 147 and 185 mm for sternum length in Polish geese. Although the mean weights in this study showed lower values than those of El-Hanoun et al. [47] in Egyptian geese (4483 g) and Polish geese (6700 g), they recorded higher values than those for Iraqi geese (2933 g) in the Kurdistan region.

The result of the stepwise discriminant analysis might indicate that these four basic measurements (chest circumference, body weight, sternum length and head length) could be more important in differentiating between the three Egyptian goose populations than other measurements. The plot of the principal component analysis (PCA) for the body measurements showed large overlaps, indicating that there are small differences in the measured variables between the three populations.

### 4.2. Mitochondrial D-Loop Analysis

The six sequenced individuals were found to be monomorphic in the amplified fragment of the D-loop, providing a single haplotype in the three Egyptian goose populations, thus indicating the same maternal origins. The low sequence divergence among the Egyptian geese from the GenBank sequence of *Anser anser* confirms that all of these samples belong to the same species (*Anser anser*), and the mtDNA D-loop sequence divergence is more suitable for the analysis of interspecies divergence than intraspecies divergence [48].

### 4.3. Microsatellite Marker Polymorphisms and Population Diversity

The average values of *N_A_*, *H_O_* and *H_E_* across ten of the studied microsatellite loci (*ZAAS006, ZAAS013, ZAAS015, ZAAS060, ZAAS038, ZAAS064, ZAAS175, ZAAS018, ZAAS152* and *ZAAS041*) in the three studied populations were comparable with those described by Li et al. [36], who recorded values of 3.7, 0.316 and 0.479 across Chinese geese, respectively. The estimated values of *N_A_*, *H_O_* and *H_E_* of the *ANS025* locus of this study were comparable with those described by Mindek et al. [49], who recorded values of 4.00, 0.43 and 0.43, respectively, in Slovak geese, while they were lower than those described by Lai et al. [50], who recorded values of 5.000, 0.496 and 0.672, respectively, in Chinese geese.

In the present study, although the *ZAAS175*, *ZAAS152* and *ZAAS041* loci showed low *N_E_* values (1.024, 1.035 and 1.089, respectively) across the three studied Egyptian populations, they showed moderate values (3.285, 1.496 and 2.273, respectively) across the Chinese population, as shown in Supplementary Table S1. The effective number of alleles (*N_E_*) is an importance diversity index as it gives an indication of the difference in allele frequency in a population. When most alleles have a low frequency and few of them have a high frequency, we expect low values of *N_E_*, *H_O_* and *H_E_* [51]. The low values of *H_O_* and *H_E_* in the current study might be attributed to the fact that most of the alleles of those loci have a low frequency and few of them have a high frequency. The relatively high and positive *F_IS_* average in this study (0.203), in addition to the seven loci showing a deficit of heterozygosity, might indicate non-random mating and inbreeding.

For interpretation of the fixation coefficient of a subpopulation within the total population (*F_ST_*), it has been suggested that values in the ranges of 0.00–0.05, 0.05–0.15, 0.15–0.25 and above 0.25 indicate little, moderate, high and very high genetic differentiation, respectively [51,52]. In the current study, the low *F_ST_* value (0.045) indicated little genetic differentiation between the three studied Egyptian populations. The estimated *F_ST_* value of the current study was lower than that measured between Chinese goose populations, which showed a value of 0.242 [26]. In this study, the high *F_ST_* value (0.343) recorded after adding the Chinese population indicated that there is high genetic differentiation between this population and the three Egyptian goose populations.

Regarding the within-population genetic diversity, although the three goose populations of this study showed lower genetic diversity parameters (*N_A_* = 3.167, *N_E_* = 1.699, *H_O_* = 0.254, *H_E_* = 0.325, *PIC* = 0.307) than those reported by Moniem et al. [28] (*N_E_* = 3.166, *H_O_* = 0.482, *H_E_* = 0.615, *PIC* = 0.565), the *F_IS_* value (0.213) was comparable to that of Moniem et al. [28], who reported a high positive value (0.224) across two Egyptian goose populations based on nine microsatellite loci. This might be attributed to the small and fragmented population size, which leads to a reduction in genetic diversity because of genetic drift and inbreeding.

Lower genetic diversity reduces individuals’ fitness and limits their ability to adapt to environmental change, consequently increasing their risk of extinction [53]. Moreover, *F_IS_* is used to obtain a deeper insight into the degree of inbreeding and endangerment potentiality and is considered an important tool to judge conservation priority [54]. Therefore, when *F_IS_* is more than 0.05 or less than 0.40, the ranges being from 0.05 to 0.15, 0.15 to 0.25 or 0.25 to 0.40, this means that the population is not in danger, is potentially endangered, minimally endangered or endangered, or is critically endangered, respectively. In this study, the Kafr El-Sheikh, Fayoum and Luxor populations showed high levels of inbreeding (0.233, 0.277 and 0.114, respectively), suggesting their potential endangerment [54].

### 4.4. Genetic Relationship and Population Structure

The close relationship between the Fayoum and Luxor populations indicated by the clustering pattern of the neighbor-joining phylogenetic tree and STRUCTURE, in addition to the low values of pairwise genetic distance (*D_A_*) and pairwise *F_ST_*, might be attributed to the high social connection between the Luxor and Fayoum governorates, where human activities, such as marketing, can shape the goose population structure, as the genetic diversity in its domestic distribution is the result factors such as geography, ecology, behavior and molecular aspects, which hierarchically interact through time and space [28]. Similarly, Moniem et al. [28] reported low genetic distance (0.18) and pairwise *F_ST_* (0.11) between two Egyptian goose populations (black variety and grey variety) based on nine microsatellite loci. The pairwise *F_ST_* value between Suchovska and Slovak geese was 0.58 [49], while it ranged between 0.003 and 0.233 in Polish geese [55].

## 5. Conclusions

The three investigated indigenous goose populations from Upper (Luxor), Middle (Fayoum) and Delta (Kafr El-Sheikh) Egypt showed a high inbreeding level and low genetic differentiation based on mitochondrial D-loop and microsatellite markers, suggesting their potential endangerment. Moreover, they showed low differentiation based on morphological measurements. The high inbreeding and low genetic and morphological differentiation could be corrected by establishing a large base population through capturing small populations with the highest genetic variation. The *ZAAS175*, *ZAAS152* and *ZAAS041* loci showed low diversity, and the *ZAAS035* locus showed a monomorphic pattern, so those loci are not recommended to be used for genotyping Egyptian geese. These results provide a basis for future phenotypic and genetic variation studies and the development of conservation strategies for domestic geese in Egypt.

## Figures and Tables

**Table 1:** Number of studied birds, villages and their latitude and longitude in each governorate.

Governorate	No. Villages	No. Birds	Latitude	Longitude
Kafr El-Sheikh	13	122	31.203548	30.550222
Fayoum	13	132	29.417017	30.712002
Luxor	12	148	25.743246	32.695547
Total	38	402		

**Table 2:** Mean ± standard error (SE) of the body measurements in domestic Egyptian goose populations.

Trait	Kafr El-Sheikh			Fayoum			Luxor			Sig.
	Mean	±	SE	Mean	±	SE	Mean	±	SE	
Head length (mm)	126.8 ^a^	±	0.50	124.6 ^b^	±	0.55	122.7 ^c^	±	0.53	**
Culmen length (mm)	70.5 ^a^	±	0.46	70.1 ^a^	±	0.51	68.5 ^b^	±	0.49	*
Bill width (mm)	25.3	±	0.12	25.6	±	0.13	25.2	±	0.13	ns
Tarsus length (mm)	103.0 ^a^	±	0.56	103.7 ^a^	±	0.61	100.1 ^b^	±	0.59	**
Chest circumference (cm)	46.5	±	0.24	45.8	±	0.27	45.8	±	0.26	ns
Sternum length (mm)	149.6 ^a^	±	0.90	145.5 ^b^	±	0.86	145.5 ^b^	±	0.81	ns
Body weight (g)	3468.9 ^a^	±	39.0	3199.1 ^b^	±	43.0	3210.6 ^b^	±	41.3	**

ns: not significant; * *p* < 0.05; ** *p* < 0.01. ^a,b,c^ Rows with different superscript letters showed significance using Duncan’s test.

**Table 3:** Summary of the stepwise selection of traits.

Step	Variables Entered	Partial R^2^	*F*-Value	*p* > *F*	Wilk’s Lambda	*p* < Lambda	Average Squared Canonical Correlation	*p* > ASCC
1	Chest circumference	0.9996	329905	<0.0001	0.000387	<0.0001	0.333	<0.0001
2	Body weight	0.2897	51.95	<0.0001	0.000275	<0.0001	0.355	<0.0001
3	Sternum length	0.1198	17.28	<0.0001	0.000242	<0.0001	0.391	<0.0001
4	Head length	0.1270	18.43	<0.0001	0.000211	<0.0001	0.414	<0.0001
5	Bill width	0.0395	5.19	0.0016	0.000203	<0.0001	0.423	<0.0001
6	Tarsus length	0.0246	3.18	0.0240	0.000198	<0.0001	0.429	<0.0001

**Table 4:** Observed (*N_A_*) and effective (*N_E_*) number of alleles, observed (*H_O_*) and expected (*H_E_*) heterozygosity and F-statistics (*F_IS_* and *F_ST_*) and polymorphism information content (*PIC*) across the three Egyptian goose populations.

Loci	*N_A_*	*N_E_*	*H_O_*	*H_E_*	*F_IS_*	*F_ST_*	*PIC*	*HWE*
*ZAAS006*	7	1.745	0.238	0.412	0.397	0.051	0.389	**
*ZAAS013*	4	2.729	0.387	0.637	0.385	0.013	0.564	**
*ZAAS015*	5	1.631	0.322	0.378	0.135	0.048	0.328	ns
*ZAAS060*	3	1.957	0.533	0.493	−0.092	0.036	0.390	ns
*ZAAS038*	5	2.748	0.454	0.621	0.273	0.047	0.606	**
*ZAAS064*	2	1.278	0.223	0.217	−0.040	0.006	0.193	**
*ZAAS175*	2	1.024	0.024	0.023	−0.012	0.001	0.023	**
*ANS025*	5	1.754	0.386	0.431	0.105	0.014	0.385	ns
*ZAAS018*	7	2.373	0.417	0.549	0.275	0.258	0.668	**
*ZAAS152*	2	1.035	0.021	0.034	0.317	0.009	0.032	**
*ZAAS041*	3	1.089	0.041	0.081	0.372	0.009	0.077	**
Mean ± SE	4.091 ± 0.212	1.760 ± 0.122	0.277 ± 0.035	0.352 ± 0.041	0.203 ± 0.060	0.045 ± 0.022	0.307 ± 0.064	
^†^ Total mean *±* SE	4.000 ± 0.204	2.134 ± 0.139	0.399 ± 0.034	0.445 ± 0.035	0.100 ± 0.042	0.343 ± 0.049	0.388 ± 0.053	

^†^ Total mean includes Chinese geese in addition to the three Egyptian populations. ns: not significant; ** *p* < 0.01.

**Table 5:** Observed (*N_A_*) and effective (*N_E_*) number of alleles, observed (*H_O_*) and expected (*H_E_*) heterozygosity and fixation coefficient of an individual within a subpopulation (*F_IS_*) per population.

Population	*N*	*N_A_*	*N_E_*	*H_O_*	*H_E_*	*F_IS_*	*HWE*
Kafr El-Sheikh	58	3.000	1.587	0.222	0.308	0.233	*
Fayoum	59	3.417	1.710	0.279	0.343	0.277	*
Luxor	56	3.083	1.800	0.261	0.323	0.114	*
Chinese	31	3.667	2.381	0.478	0.492	−0.003	*
Mean ± SE	173	3.167 ± 0.216	1.699 ± 0.117	0.254 ± 0.034	0.325 ± 0.040	0.213 ± 0.047	
^†^ Total mean ± SE	204	3.292 ± 0.208	1.869 ± 0.133	0.310 ± 0.034	0.367 ± 0.036	0.159 ± 0.042	

^†^ Total mean includes Chinese geese in addition to the three Egyptian populations. * *p* < 0.05.

**Table 6:** Reynolds genetic distance (above diagonal) and pairwise *F_ST_* (below diagonal) estimates for the 11 microsatellite loci between the four studied goose populations.

	Fayoum	Kafr El-Sheikh	Luxor	Chinese
**Fayoum**		0.056 (0.061) *	0.023 (0.029)	1.532 (1.543)
**Kafr El-Sheikh**	0.035		0.071 (0.077)	1.648 (1.660)
**Luxor**	0.022	0.044		1.680 (1.691)
**Chinese**	0.383	0.404	0.406	

* Nei’s genetic distance is presented above the diagonal in brackets.

## Data Availability

The underlying research data can be obtained from the corresponding author, who can be reached by email: sherif.ramadan@fvtm.bu.edu.eg.

## Supplementary Materials

The following are available online at https://www.mdpi.com/article/10.3390/ani11113106/s1, Figure S1: Morphological body measurements taken using diagonal calipers and a metric ruler. Table S1: Observed (*N_A_*) and effective (*N_E_*) number of alleles, observed (*H_O_*) and expected (*H_E_*) heterozygosity, inbreeding coefficient (*F_IS_*), and polymorphism information content (*PIC*) across the Chinese goose populations.
