# Supplementary material for: Genetic and Phenotypic Characterization of Domestic Geese (Anser anser) in Egypt
Source: Animals (Basel). 2021 Oct 30;11(11):3106. doi: 10.3390/ani11113106 (PMC8614349; doi:10.3390/ani11113106)
Supplement: Supplementary file 1 [file animals-11-03106-s001.zip › Supplementary Figures S1.pdf]

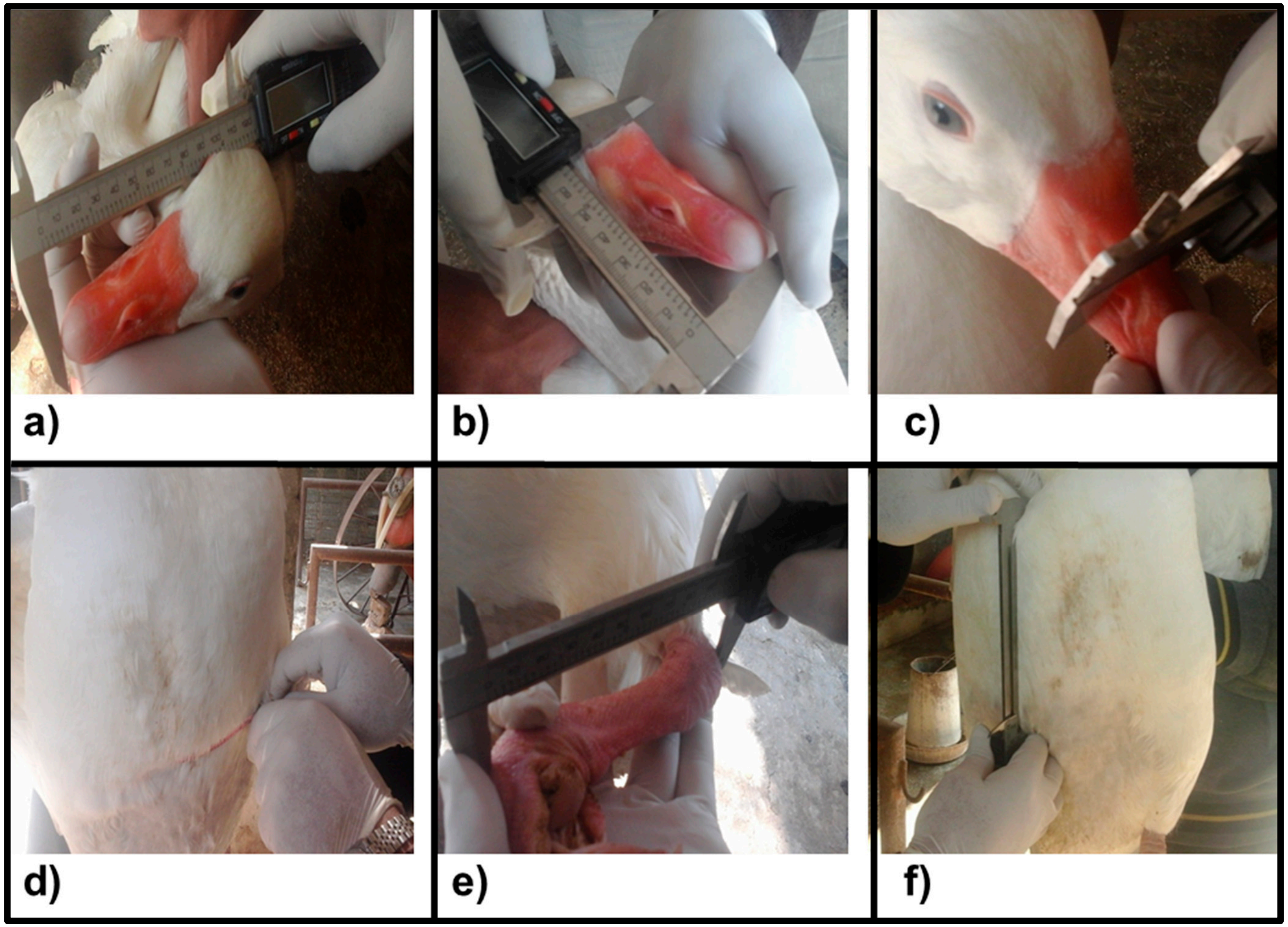

Supplementary Figure S1. Morphological body measurements taken using diagonal calipers and a metric ruler, (**a**) head length (**b**) culmen length (**c**) bill length (**d**) chest circumference (**e**) tarsus length (**f**) sternum length
