# Supplementary material for: Genetic and Phenotypic Characterization of Domestic Geese (Anser anser) in Egypt
Source: Animals (Basel). 2021 Oct 30;11(11):3106. doi: 10.3390/ani11113106 (PMC8614349; doi:10.3390/ani11113106)
Supplement: Supplementary file 1 [file animals-11-03106-s001.zip › Table_S1.pdf]

Supplementary Table S1. Observed (N_A) and effective (N_E) number of alleles, observed (H_O), and expected (H_E) heterozygosity, inbreeding coefficient (F_{IS}) and polymorphism information content (*PIC*), across the Chinese goose populations

Locus	N_A	N_E	H_O	H_E	F_{IS}	*PIC*	*HWE*
ZAAS006	4	2.423	0.857	0.598	-0.459	0.503	ns
ZAAS013	5	1.405	0.250	0.294	0.133	0.278	**
ZAAS015	5	2.469	0.643	0.606	-0.080	0.541	ns
ZAAS060	2	1.412	0.355	0.297	-0.216	0.249	ns
ZAAS038	5	3.263	0.792	0.708	-0.141	0.635	ns
ZAAS064	2	1.923	0.533	0.488	-0.111	0.365	ns
ZAAS175	4	3.285	0.645	0.707	0.073	0.633	ns
ANS025	2	1.811	0.226	0.455	0.496	0.348	**
ZAAS018	7	5.814	0.480	0.845	0.420	0.804	***
ZAAS152	2	1.496	0.355	0.337	-0.071	0.277	ns
ZAAS041	5	2.273	0.600	0.569	-0.071	0.513	ns
Mean ± SE	3.909±0.513	2.507±0.387	0.521±0.063	0.537±0.055	-0.003±0.083	0.468±0.054	

ns: not significant, ** $p < 0.05$, *** $p < 0.01$
